# G2 checkpoint abrogation and checkpoint kinase-1 targeting in the treatment of cancer

**DOI:** 10.1038/sj.bjc.6604208

**Published:** 2008-01-29

**Authors:** N Bucher, C D Britten

**Affiliations:** 1 Division of Hematology and Oncology, Department of Medicine, David Geffen School of Medicine, University of California, Los Angeles, CA, USA

**Keywords:** checkpoint, cell cycle, G2, Chk1

## Abstract

Rigorous quality control steps, termed checkpoints, tightly regulate progression through the cell cycle. DNA-damaging chemotherapy and radiation activate functional cellular checkpoints. These checkpoints can facilitate DNA repair and promote cell death in unrepaired cells. There are at least three DNA damage checkpoints – at G1/S, S, and G2/M – as well as a mitotic spindle checkpoint. Most cancer cells harbour mutations in tumour suppressors and/or oncogenes, which impair certain cell checkpoints. Inhibiting the remaining cell checkpoints – particularly after exposure of cancer cells to chemotherapy and/or radiation – allows cell death, a strategy now being employed in cancer therapeutics. With our increasing knowledge of cell cycle regulation, many compounds have been developed to inhibit specific checkpoint components, particularly at the G2/M transition. One such target is checkpoint kinase-1 (Chk1). We review here the molecular framework of the cell cycle, the rationale for targeting Chk1, the preclinical concepts related to the development of Chk1 inhibitors, and the efficacy and safety results from Chk1 inhibitors now in phase I/II trials.

The cell cycle is organised into a series of dependent pathways, whereby the initiation of each event is dependent upon successful completion of previous events. In this way, replicating cells traverse the four distinct phases of the cell cycle consecutively: G1 followed by S, followed by G2 and, finally, M. This ordered progression is guarded by checkpoints capable of delaying the cell cycle in response to intra- or extracellular stressors. As part of the cell cycle surveillance system, the DNA damage and spindle checkpoints protect the cell from genomic instability. Checkpoints are important quality control measures that ensure the proper sequence of cell cycle events and allow cells to respond to DNA damage.

Increasingly, checkpoint inhibition has become an area of novel drug development. In the setting of DNA damage, checkpoint inhibition leads to genomic instability, and subsequent cell death. The first checkpoint, found at the G1/S transition, is compromised in many malignant cells, due to mutations in various tumour suppressor genes, including retinoblastoma protein (Rb) and p53. Cells deficient in the G1 checkpoint are dependent on the S and G2 checkpoints for DNA repair. Checkpoint kinase-1 (Chk1) is an active transducer kinase at both the S and G2 checkpoints, rendering it a target for rational anticancer drug development. In the presence of DNA damage, Chk1 inactivation abrogates G2 arrest, resulting in preferential cancer cell death (Chen *et al*, 2003).

This article serves to review the (1) current molecular pathways comprising the cell cycle checkpoint machinery, (2) inhibition of Chk1 as an effective means of abrogating G2 arrest, and (3) current Chk1 inhibitors in use in phase I clinical trials.

## MOLECULAR COMPONENTS OF THE DNA DAMAGE CHECKPOINTS

**Figure 1:**
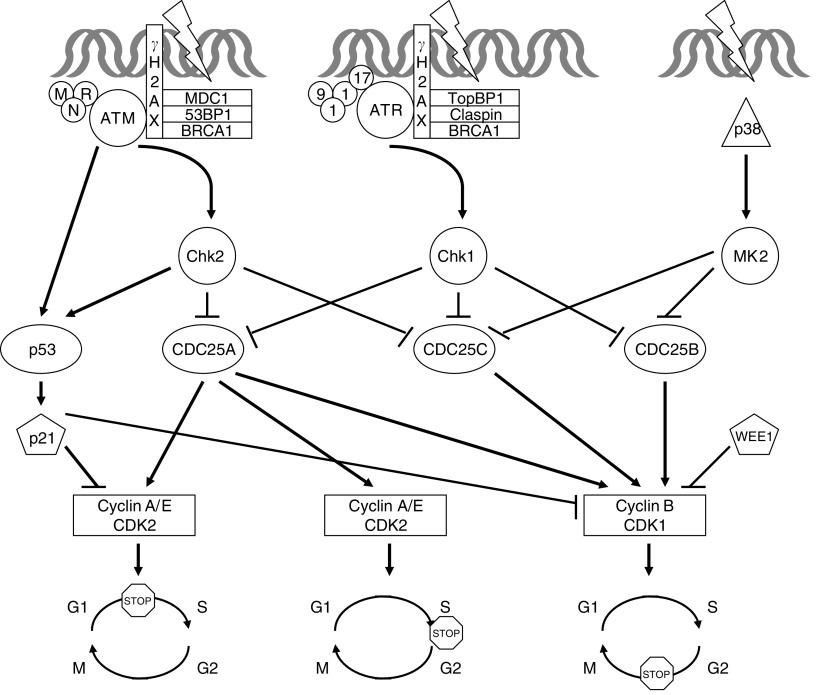
Cell cycle checkpoint pathways. Once DNA damage is identified with the aid of sensors, the checkpoint transducers ATM and ATR undergo conformational change and/or localisation, resulting in their activation. Together with their mediators, ATM and ATR activate a series of downstream molecules, including the checkpoint transducer kinases. Checkpoint kinase-2 and Chk1 inactivate CDC25 phosphatases, culminating in cell cycle arrest.

Components of the checkpoint mechanism include sensors, mediators, transducers, and effectors, which work cooperatively in different phases of the cell cycle (Sancar *et al*, 2004) (Figure 1). The phosphatidylinositol 3-kinase-related kinases ATM (ataxia–telangiectasia mutated) and ATR (ATM and Rad3-related) are transducers that coordinate the initiation, amplification, and activation of the checkpoint through phosphorylation of many different targets. Although ATM and ATR are classified as transducers, they are capable of recognising DNA damage. Ataxia–telangiectasia mutated is activated by DNA damage from ionising radiation, whereas ATR is activated by DNA damage and DNA replication stress. In the case of ATM, DNA double-strand breaks (DSBs) induce ATM homodimer dissociation. The resultant ATM monomers are recruited to sites of DSBs, with the aid of the putative sensor MRN complex, comprised of Mre11, Rad50, and Nbs1 (Van Den Bosch *et al*, 2003). In the case of ATR, short sequences of single-strand breaks are generated from DSBs, and coated with replication protein A (RPA). Replication protein A-coated DNA recruits ATR together with its interacting protein ATRIP (Zou and Elledge, 2003). Full activation of the ATR/ATRIP complex and successful checkpoint function requires loading of the sensor Rad17 and 9-1-1 (Rad9, Rad1, and Hus1) complexes onto DNA (Zou *et al*, 2002).

Mediators are proteins that facilitate the activation of ATM and ATR substrates. In one model, ATM phosphorylates histone H2AX, flanking the sites of DNA damage. Proteins including mediator of DNA damage checkpoint 1 (MDC1), p53-binding protein (53BP1), and BRCA1 accumulate at phosphorylated H2AX (*γ*H2AX), culminating in Chk2 activation (Canman, 2003). In another model, ATR interacts with the mediator TopBP1 to phosphorylate a number of proteins, including H2AX (Liu *et al*, 2006). The interaction of ATR with TopBP1, and its downstream mediator claspin, results in recruitment and phosphorylation of BRCA1 and subsequent activation of Chk1.

Checkpoint kinase-1 and Chk2 are the checkpoint transducer kinases that function downstream in the DNA-damage checkpoint signalling pathway. Although structurally dissimilar, Chk1 and Chk2 are serine/threonine kinases that serve as functional analogues (Bartek *et al*, 2001). Checkpoint kinase-2, expressed throughout the cell cycle, is activated in the presence of DNA damage (Lukas *et al*, 2001). In contrast, Chk1, preferentially expressed during S and G2, has constitutive activity that is amplified in the presence of DNA damage (Zhao *et al*, 2002). Ataxia–telangiectasia mutated phosphorylates Chk2 at threonine 68, and ATR phosphorylates Chk1 at serines 317 and 345. Significant crosstalk exists between the ATM/Chk2 and ATR/Chk1 pathways (Gatei *et al*, 2003). Although Chk1 and Chk2 have overlapping roles in checkpoint signalling, only Chk1 is indispensable for mammalian survival (Liu *et al*, 2000).

Another transducer kinase, downstream from the stress–response p38 MAPK pathway and named MAPKAP kinase-2 (MK2), is directly involved in phosphorylating effectors CDC25B and C, and in maintaining G1, S, and G2 checkpoints triggered by UV-induced DNA damage (Manke *et al*, 2005). MAPKAP kinase-2 is activated by cisplatin, camptothecin, and doxorubicin, and the MK2 response is essential for the survival of p53-deficient cells following exposure to these agents (Reinhardt *et al*, 2007).

Together, the proximal transducers ATM and ATR and the distal transducers Chk1, Chk2, and MK2 phosphorylate a variety of effector molecules, such as p53 and CDC25 phosphatases, culminating in cell cycle arrest. For the purpose of this review, an understanding of CDC25 phosphatases is key. The three CDC25 isoforms – A, B, and C – are active in different phases of the cell cycle. CDC25 phosphatases remove inhibitory phosphate groups from cyclin/cyclin-dependent kinase (CDK) complexes, promoting cell cycle progression. In response to DNA damage, the checkpoint kinases phosphorylate CDC25 phosphatases, resulting in CDC25 inactivation through either ubiquitin-mediated degradation or cytoplasmic sequestration. In this manner, the checkpoint kinases serve as negative regulators of the CDC25 phosphatases.

## CELL CYCLE CHECKPOINTS

Although there is redundancy in checkpoint signalling, the relative contribution of individual checkpoint transducers and effectors varies during the course of the cell cycle, as described below.

### G1 checkpoint

The G1 checkpoint is the first defence against genomic stress in cycling cells. In response to DNA damage, the G1 checkpoint prevents cells from entering the S phase by inhibiting the initiation of DNA replication. At this checkpoint, Chk2 is activated by ATM to phosphorylate CDC25A phosphatase (Massague, 2004), preventing activation of cyclin E(A)/CDK2 (Mailand *et al*, 2000) and temporarily halting the cell cycle. It has been proposed that G1 arrest is sustained by ATM/Chk2-mediated phosphorylation of murine double minute protein and p53, resulting in p53 stabilisation (Takai *et al*, 2002) and accumulation. p53 activates transcription of the CDK inhibitor p21, which in turn inhibits cyclin E(A)/CDK2 (Harper *et al*, 1993) and preserves the association of Rb with E2F. Reports that Chk2-null mouse embryo fibroblasts manifest p21 induction and G1 arrest upon exposure to radiation have called into question the role of Chk2 in DNA damage-induced G1 arrest (Jack *et al*, 2002). Instead, Chk2 appears to be necessary for p53-mediated apoptosis (Jack *et al*, 2002). While interactions between Chk2 and p53 are under investigation, most human cancers are deficient in p53 (Kastan *et al*, 1991). As a result, cancer cells accelerate through the cell cycle until they meet the remaining barriers of the cell cycle, namely, the S and G2 checkpoints.

### S-phase checkpoint

The S-phase checkpoint serves to address both DNA replication errors and DNA damage incurred during S phase (Bartek *et al*, 2004). Ionising radiation may transiently slow DNA synthesis through two parallel pathways: ATR(ATM)/Chk1(Chk2)/CDC25A/CDK2 and ATM/NBS1/MRE11/structural maintenance of chromosome 1 (SMC1) (Falck *et al*, 2002). In the first pathway, DNA damage invokes ATR/Chk1 and ATM/Chk2, resulting in CDC25A degradation, thereby inhibiting cyclin E(A)/CDK2 (Bartek *et al*, 2004) and progression through S phase. Checkpoint kinase-1 is thought to be the primary S-phase checkpoint kinase, with Chk2 playing a supportive role. This is supported by studies with siRNAs targeting Chk1 and Chk2, demonstrating that downregulation of Chk1, but not Chk2, abrogates camptothecin- or 5-fluorouracil-induced S-phase arrest (Xiao *et al*, 2003). In the second pathway, the sensor MRN complex recruits ATM to sites of DNA damage with the help of the MDC1 (Watrin and Peters, 2006). Once localised to damaged DNA, ATM phosphorylates SMC1, a component of the cohesin complex thought to function in DNA repair. The mechanism by which SMC1 slows S-phase progression is under study.

### G2 checkpoint

Cells that have either incurred DNA damage in G2 phase, or that have escaped the G1 and S checkpoints despite earlier genomic insults, are stalled at the G2 checkpoint. At G2, Chk1 is activated by ATR to phosphorylate CDC25A, -B, and -C (Boutros, 2006), preventing cyclin B/CDK1 activation and resulting in G2 arrest. Another mechanism of G2 arrest is provided by stress-induced activation of p38 MAPK/MK2 and subsequent inactivation of CDC25B/C, as described earlier (Manke *et al*, 2005; Reinhardt *et al*, 2007). By inducing the transcription of p21 and other proteins, p53 also plays a role in the G2 checkpoint (Taylor and Stark, 2001).

## G2 ABROGATION AS AN ANTICANCER STRATEGY

Cancer cells are dependent on the S and G2 checkpoints for repair of DNA damage, due to the presence of defective G1 checkpoint mechanisms. Because the S-phase checkpoint facilitates slowing, rather than arrest, of the cell cycle, a cancer cell harbouring DNA damage may progress through the S checkpoint, only to halt at the G2 checkpoint. Thus, the G2 checkpoint is a key guardian of the cancer cell genome, and it has emerged as an attractive therapeutic target for anticancer therapy. G2 abrogation prevents cancer cells from repairing DNA damage, forcing them into M phase and the so-called ‘mitotic catastrophe’ and apoptosis.

The ideal G2 checkpoint abrogator would be selective, targeting a molecule not involved in G1 checkpoint or S-phase checkpoint or, if involved, in a nonredundant fashion (Kawabe, 2004). Candidate targets for G2 abrogation are discussed below.

### ATM/ATR inhibition

Ataxia–telangiectasia mutated and ATR activate pathways involved in cell cycle checkpoints, apoptosis, and DNA repair; therefore, they are not specific G2 checkpoint abrogators. Nonetheless, caffeine, which has many molecular effects, including ATM and ATR inhibition, has been shown to potentiate the cytotoxicity of nitrogen mustard by disrupting the G2 checkpoint, inducing damaged cells to undergo mitosis before properly repairing lesions in their DNA (Lau and Pardee, 1982). The clinical toxicity of caffeine at millimolar concentrations prevents further clinical evaluation (Stewart *et al*, 1987). A less toxic derivative of caffeine, pentoxifylline, has been tested in clinical trials; however, its effects on cell biology are also nonspecific (Kawabe, 2004). More specific ATM inhibitors are currently in development (Hickson *et al*, 2004).

### Manipulation of CDC25 and WEE1

Another strategy to abrogate the G2 checkpoint is to activate CDC25C phosphatase, in conjunction with DNA damage. Activating this phosphatase results in dephosphorylation and activation of cyclin B/CDK1, causing cell cycle progression to M phase. To date, no such activators have been developed. An alternative method of G2 abrogation is the inhibition of WEE1, a protein that opposes CDC25 activity by phosphorylating and inactivating cyclin/CDK complexes. As an example, the WEE1 inhibitor PD0166285 has demonstrated G2 checkpoint inhibition in preclinical models (Hashimoto *et al*, 2006).

### MK2 inhibition

The p38 MAPK/MK2 pathway has been implicated in several cancer cell pathways, from those related to inflammation, growth, replication, apoptosis, angiogenesis, and metastasis. More recently, this pathway has been found to be a regulator of checkpoint controls, particularly at the G2/M transition (Manke *et al*, 2005). MK2-depleted p53-deficient cells cause not only abrogation of the CDC25A-mediated S-phase checkpoint after cisplatin treatment, but also loss of the CDC25B-mediated G2/M checkpoint following doxorubicin (Reinhardt *et al*, 2007). As such, an MK2 inhibitor may sensitise cancer cells to cytotoxic agents. However, in one study of Chk1 and MK2 downregulation with siRNA, suppression of MK2 did not abrogate chemotherapy-induced cell cycle arrest, and it appeared to antagonise checkpoint abrogation provided by suppression of Chk1 (Xiao *et al*, 2006).

### HSP90 inhibition

An indirect and nonspecific method of checkpoint abrogation is provided by inhibition of the molecular chaperone heat shock protein-90 (HSP-90). In preclinical studies, the HSP-90 inhibitor 17-AAG has been shown to deplete Chk1, an HSP-90 client (Arlander *et al*, 2003). Likewise, G2/M abrogation was seen when 17-AAG was combined with SN38 in p53-deficient cells (Tse *et al*, 2005) and when combined with irradiation in human lung cancer cells. The HSP-90 inhibitor 17-AAG is in clinical development, along with many other HSP-90 inhibitors.

### Chk1 inhibition

Perhaps the most relevant approach to G2 checkpoint abrogation is the inhibition of Chk1 kinase. Checkpoint kinase-1 is a key element in the DNA damage response pathway and plays a crucial role in the S-phase checkpoint and G2 checkpoint, largely mediated by CDC25A. In addition, Chk1 is required for mitotic spindle checkpoint function (Zachos *et al*, 2007). The spindle checkpoint delays anaphase until proper chromosomal attachment and segregation, and depletion of Chk1 induces chromosomal instability. In this manner, Chk1 inhibitors are capable of not only enhancing the efficacy of DNA-damaging agents that cause S or G2 arrest, but also potentiating antimitotic activity.

Use of DNA-damaging agents or antimitotics, in combination with a Chk1 inhibitor, not only confers enhanced tumour kill, but also may eliminate cell cycle-mediated drug resistance. Depending on the cell's position in the cell cycle and on the particular checkpoints activated, a cell may demonstrate a relative insensitivity to a chemotherapeutic agent (Shah and Schwartz, 2001). Appropriate scheduling and sequencing of cell cycle checkpoint inhibitors could thus overcome the limited efficacy of cytotoxic drugs.

Several Chk1 inhibitors have been studied in the laboratory over the past decade, some of which have been reviewed previously (Kawabe, 2004; Tse *et al*, 2007a). Examples of compounds that are in advanced preclinical and/or early clinical development are listed in Table 1, and potential biomarkers of Chk1 inhibition are presented in Table 2.

## CHK1 INHIBITORS IN THE CLINIC

### UCN-01

7-Hydroxystaurosporine (UCN-01) has multiple cell cycle effects including inhibition of Chk1 and MK2 with IC_50_ values of 7 nM (Kawabe, 2004) and 95 nM (Reinhardt *et al*, 2007), respectively. The compound UCN-01 has demonstrated *in vitro* synergy with many chemotherapeutic agents, leading to multiple clinical trials employing UCN-01 in combination (Tse *et al*, 2007a). The clinical utility of UCN-01 may be limited by its avid plasma-protein binding and dose-limiting hyperglycaemia (Kortmansky *et al*, 2005).

### CBP501

CBP501 is a synthetic peptide that was generated to suppress phosphorylation of CDC25C at serine 216, to prevent cytoplasmic sequestration. Subsequently, the inhibitory effects of CBP501 were found to be most pronounced against MK2 (IC_50_ 0.9 μM), C-Tak1 (IC_50_ 1.4 μM), and Chk1 (IC_50_ 3.4 μM) (Sha *et al*, 2007). *In vitro* and *in vivo*, CBP501 increased the anticancer activity of cisplatin and bleomycin. In a single-agent phase I clinical trial, patients received doses of 0.9–7.2 mg m^−2^ i.v. weekly for 3 weeks with 1 week off. Preliminary results indicate the main toxicity to be grade 2 allergic reaction, with no dose-limiting toxicity reported to date. Decreased phosphorylation of CDC25C at serine 216 was demonstrated in peripheral blood lymphocytes from 7 of 12 patients evaluated, suggesting biologic activity at the G2 checkpoint (Gordon *et al*, 2006). A combination phase I study of CBP501 and cisplatin is currently underway.

### XL844

XL844 (EXEL9844) is a novel and specific inhibitor of both Chk1 and Chk2, with IC_50_ values of 2.2 and 0.2 nM, respectively (Matthews, 2006). In preclinical studies, it reversibly and competitively inhibited Chk1 at the ATP-binding site. In an *in vitro* CML model, XL844 abrogated the G2 checkpoint activated by daunorubicin-induced DNA damage, as indicated by CDK1 activation and an increase in phosphohistone H3 (a marker of mitotic entry). In a CML nude mice survival model, the combination of XL844 and daunorubicin caused a significant increase in median survival time. In a panel of multiple solid tumour cell lines, XL844 had little effect as a single agent, but substantially increased the cytotoxicity of gemcitabine (Matthews *et al*, 2007). XL844 was shown to affect both the S and G2 checkpoints by blocking gemcitabine-induced DSBs and CDC25A phosphorylation, inducing premature mitotic entry. XL844 also resulted in an increase in gemcitabine-induced *γ*H2AX. In a pancreatic tumour xenograft model, increasing doses of XL844 enhanced gemcitabine's antitumour activity, without an increase in toxicity. XL844 was the first specific Chk1/2 inhibitor to enter phase I clinical trials, in patients with refractory chronic lymphocytic leukaemia; however, this trial closed due to slow enrollment. Currently, a phase I dose-escalation study of XL844 alone and in combination with gemcitabine is underway.

### PF-00477736

PF-00477736 is a potent, selective ATP-competitive diazapinoindolone that inhibits Chk1 with a *K*_i_ of 0.49 nM. (Anderes *et al*, 2006). The ability of PF-00477736 to abrogate the G2 checkpoint in camptothecin-treated cells was demonstrated by an increase in phosphohistone H3 levels and by an increase in a sub-G1 population. PF-00477736 also induced checkpoint abrogation in gemcitabine-treated cells, as demonstrated by a number of molecular endpoints, including decreased activation of Chk1 at serine 345, increased *γ*H2AX, and increased apoptosis. PF-00477726 enhanced the cytotoxicity of gemcitabine, irinotecan, and carboplatin, with selectivity for p53-defective cancer cell lines compared with p53-competent cells. In colon cancer xenograft models, PF-00477736 enhanced the activity of gemcitabine and irinotecan. On the basis of these and other studies, PF-00477736 is currently being evaluated in a phase I clinical trial in combination with gemcitabine.

PF-00477736 has also been shown to enhance the antitumour activity of docetaxel, an antimicrotubule agent, indicating a role for Chk1 in the mitotic spindle checkpoint (Hallin *et al*, 2007). *In vitro*, PF-00477736 abrogated docetaxel-induced G2/mitotic arrest, resulting in a more effective induction of apoptosis than produced by docetaxel alone. PF-00477736 also significantly potentiated the activity of docetaxel in Colo205 xenografts by enhancing tumour regression and prolonging survival, without added systemic toxicity. Treatment with PF-00477736 also modulated spindle checkpoint downstream effectors cyclin B, securin, BubR1, and Aurora.

### AZD7762

Another potent and selective Chk1 inhibitor that abrogates the G2 checkpoint, AZD7762, has been shown to inhibit Chk1 at an IC_50_ of 5 nM in an HT29 cell-based assay (Ashwell, 2007). *In vitro*, treatment with AZD7762 resulted in a reduction in the concentration of DNA-damaging agents required to inhibit tumour cell growth by 50 and 100% (i.e., reduction in GI50 and GI100 values). The effects of AZD7762 were more pronounced in p53 mutant cell lines. In H460 mouse xenograft models, AZD7762 potentiated both the efficacy of gemcitabine and irinotecan, causing tumour growth delays of >10-fold and >3-fold, respectively. Investigators confirmed checkpoint pathway modulation by evaluating surrogate markers in tumour tissue treated with AZD7762, demonstrating decreased autophosphorylation of Chk1 at serine 296, and increased *γ*H2AX. In contrast to PF-00477736, AZD7762 produced increased phosphorylation of Chk1 at serine 345. A combination phase I study of AZD7762 and irinotecan is currently underway.

## CONCLUSION

Recognising that cancer cells are more dependent on the G2 checkpoint for DNA damage repair than normal cells, G2 checkpoint abrogation is being investigated as a means of enhancing the therapeutic index of cytotoxic agents. Although at least two phase I trials were performed using Chk1 inhibitors as single agents, preclinical evidence suggests that G2 checkpoint abrogation will be most successful when Chk1 inhibitors are used in combination with chemotherapy or radiation therapy. *In vitro*, Chk1 inhibitors increase the anticancer activity of chemotherapy, demonstrating the potential to sensitise resistant cells. In limited xenograft models, this enhanced anticancer activity is not associated with increased toxicity. The results of ongoing clinical trials will determine whether G2 checkpoint abrogation changes the risks and benefits of chemotherapy.

While this review focuses on Chk1 inhibitors that promote cell cycle progression, other investigators are developing specific CDK inhibitors that inhibit cell cycle progression (Shapiro, 2006). Although seemingly disparate approaches, each exploits cancer cell mutations and repair mechanisms to achieve therapeutic benefit. A common challenge will be determining the proper combination, and sequence, of targeted cell cycle drugs and cytotoxic therapy. Efforts have long been underway and, as reviewed here, several Chk1 inhibitors offer promise.

## Figures and Tables

**Table 1:** Examples of Chk1 inhibitors

**Compound**	**Chemistry**	**Manufacturer**	***In vitro* Chk1 IC_50_ (in nM)**	**Other targets (IC_50_ in nM)**	**Phase of development**	**References**
UCN-01	Staurosporine derivative	Kyowa (Tokyo, Japan)	7	PKC (4.1), MK2 (95)	Phase I/II	See text
Series of compounds	Tricyclic pyrazoles	Abbott (Abbott Park, IL, USA)	0.4–24		Preclinical	Tao *et al* (2007a)
Series of compounds	Macrocyclic ureas	Abbott	3–15		Preclinical	Tao *et al* (2007b)
Series of compounds	Granulatimide analogues	Laboratoire SEESIB (Aubiere, France)	27–33		Preclinical	Conchon *et al* (2007)
CHIR-124	Benzimidazole quinolinone	Chiron (Emeryville, CA, USA)	0.3	Chk2 (9)	Preclinical	Tse *et al* (2007b)
CBP501	Peptide	CanBas (Numazu, Japan)	3400	MK2 (900), cTak1 (1400)	Phase I	Gordon *et al* (2006); Sha *et al* (2007)
XL844	Undisclosed	Exelixis (South San Francisco, CA, USA)	22	Chk2 (0.2)	Phase I	Matthews (2006); Matthews *et al* (2007)
PF-00477736	Diazapinoindolone	Pfizer (La Jolla, CA, USA)	(*K*_i_ 0.49) (EC_50_ 45)	Chk2 (*K*_i_ 47), CDK1 (*K*_i_ 9900)	Phase I	Anderes *et al* (2006); Hallin *et al* (2007)
AZD7762	Undisclosed	AstraZeneca (Waltham, MA, USA)	5	Chk2 (<10)	Phase I	Ashwell (2007)

CDK1=cyclin-dependent kinase-1; Chk1=checkpoint kinase-1; Chk2=checkpoint kinase-2; MK2=MAPKAP kinase-2; UCN-01=7-hydroxystaurosporine.

**Table 2:** Potential biomarkers of Chk1 inhibition

**Biomarker**	**Rationale**	**Expected outcome^a^**
Chk1 phosphoserine 296	Chk1 autophosphorylation site	↓
Chk1 phosphoserine 345	Chk1 activation site	↑ or ↓, depending on properties of specific inhibitor
*γ*H2AX	Activated at sites of DNA damage	↑
Phosphohistone H3	Marker of mitotic entry	↑
CDC25C phosphoserine 216	Negative regulation of CDC25C phosphatase, causing G2 arrest	↓

Chk1=checkpoint kinase-1.

a Expected outcome with addition of Chk1 inhibitor to chemotherapy-treated cells.
